# Different Genotypes of the Rare and Threatened Moss *Physcomitrium eurystomum* (Funariaceae) Exhibit Different Resilience to Zinc and Copper Stress

**DOI:** 10.3390/plants14020224

**Published:** 2025-01-15

**Authors:** Djordje P. Božović, Marija V. Ćosić, Vladislav Kolarčik, Michal Goga, Claudio Varotto, Mingai Li, Aneta D. Sabovljević, Marko S. Sabovljević

**Affiliations:** 1 Institute of Botany and Botanical Garden “Jevremovac”, Faculty of Biology, University of Belgrade, Takovska 43, 11000 Belgrade, Serbia; 2 Biodiversity, Ecology and Environment Area, Research and Innovation Centre of Fondazione Edmund Mach, Via E. Mach 1, 38098 San Michele all’Adige, Italy; 3 Department of Plant Biology, Institute of Biology and Ecology, Faculty of Science, Pavol Jozef Šafárik University in Košice, Mánesova 23, 040 01 Košice, Slovakia; 4 NBFC—National Biodiversity Future Center, 90133 Palermo, Italy; 5 Center of Plant Biotechnology and Conservation (CPBC), Takovska 43, 11000 Belgrade, Serbia

**Keywords:** bryophyte, survival, metal, tolerance, morphogenesis, flow cytometry

## Abstract

The funarioid moss species *Physcomitrium eurystomum*, which is threatened with extinction, was the subject of this study. The riparian habitat type of this species is often under the influence of contaminated water, and, therefore, we tested the influence of selected potentially toxic elements (PTEs), namely zinc and copper, on the development, physiological features, and survival of the species on two different accessions (German and Croatian). The results obtained showed the different resilience of the two accessions to the PTEs. Flow cytometry analyses revealed that the two accessions differ significantly in terms of genome size. However, the different amplitude of resilience to the tested PTEs, the divergence in physiological responses, and survival within two accessions of the same species are confirmed, as well as the dissimilarity of their genome size, likely associated with ploidy level difference and possibly distinct hybrid origin.

## 1. Introduction

The response of plants to various stress factors is usually studied at the species level. However, the amplitude of responses to stressors within a population and/or between different accessions is rarely studied. In mosses, there are even fewer such studies, although researchers are generally aware of the different responses of the same species representatives to certain stressors (e.g., stress from potentially toxic elements, [1]).

*Physcomitrium eurystomum* Sendtn. is a moss species from the Funariaceae family, with a primarily temperate distribution across Europe and Asia, though it has also been documented outside its main range. It is an ephemeral species that thrives on muddy soils along streambanks and lake shores with fluctuating water levels [2,3]. This acrocarpous moss, characterized by an annual shuttle life strategy, a short life cycle, and frequent sporophyte production, typically emerges twice a year in favorable habitats during wet seasons (spring and autumn in temperate climates) when flooding and changes in water levels expose muddy banks.

The overall European population of *P. eurystomum* is assessed as threatened with extinction and is listed as vulnerable (VU), while within the European Union, the species is red-listed and considered endangered (EN) [4]. The species is red-listed nationally in Austria, Great Britain, and Hungary, where it is considered as EN, and also in the Czech Republic, Estonia, Germany, Slovakia, and Switzerland, where it is considered as VU [5]. Furthermore, the species is considered to be at some risk of extinction in Belarus, Belgium, and the Netherlands [5]. In Serbia, *P. eurystomum* is localized and listed as EN [6]. In Slovenia, it has not been observed since 1910 [7], while it has recently been reported in Montenegro [8].

Since *P. eurystomum* expresses shuttle life form and is considered to be an extinction risk, our main hypothesis states that water contamination in its aquatic habitats, i.e., the content of potentially toxic elements (PTE), among other environmental challenges, could significantly affect its survival, growth, and maturation. This has already been shown for other aquatic and semi-aquatic plants [9,10,11].

With the aim of investigating the effects of PTE stress and documenting whether there are differences in the PTE stress resilience of threatened moss *P. eurystomum*, two accessions of this species were tested for two selected metals, namely zinc, and copper, as these have previously been applied in such studies on bryophytes, and are both essential for the normal development and function of the plant organism [12]. The concentrations and the zinc and copper acetate salts were selected based on previous studies on bryophytes (e.g., [13,14]). It is assumed that exogenously added acetate ions have no physiological side effects for plant organisms, as they can be well equilibrated in plant cell homeostasis [15] and therefore the effects of zinc or copper could be directly documented.

Considering that this threatened species inhabits riparian habitats and muddy soils of periodically dry standing water bodies and sites that are frequently affected by sudden floods, we wanted to test whether potentially toxic elements (namely zinc and copper) can affect the development and survival of this species.

Therefore, the following questions were addressed: (1) Can short-term flooding with PTE-contaminated water affect the development and survival of *P. eurystomum*? (2) Are there differences in physiological responses to PTEs in two different accessions? (3) Are there differences in genome size between the two accessions?

Therefore, two potentially different accessions/geotypes/genotypes, namely one from Germany (DE) and one from Croatia (HR), were treated the same way, as different physiological responses are assumed to confirm the variation in stress responses in the same species but different accessions/geotypes/genotypes.

## 2. Results

The main effect of genotype (G) did not have a significant influence on the index of multiplication after 2 h of zinc acetate treatment, while zinc acetate concentration (C) and the interaction between genotype and concentration (G × C) showed significant effects (*p* < 0.01 and *p* < 0.001, respectively) (Table 1). In the case of 24 h treatments, the main effect of genotype (G) and interaction (G × C) significantly affected the index of multiplication (*p* < 0.01 and *p* < 0.001, respectively) (Table 1). Regarding the diameter of the secondary protonema patch after 2 h of exposure, the main effects of both, genotype (G) and zinc acetate concentration (C) showed significant effects (*p* < 0.001), while the interaction (G × C) exhibited no significant effects (Table 1). After 24 h of exposure, the main effects of genotype (G), zinc acetate concentration (C), and their interaction (G × C) all significantly affected the diameter of the secondary protonema patch (*p* < 0.001) (Table 1). Total chlorophyll content was not significantly affected by genotype (G), zinc acetate concentration (C), or their interaction (G × C), at either time of exposure (Table 1), suggesting that the chlorophyll content was not affected by the treatment of different concentrations of zinc acetate with different exposure times for both accessions of *P. eurystomum*.

**Figure 1:**
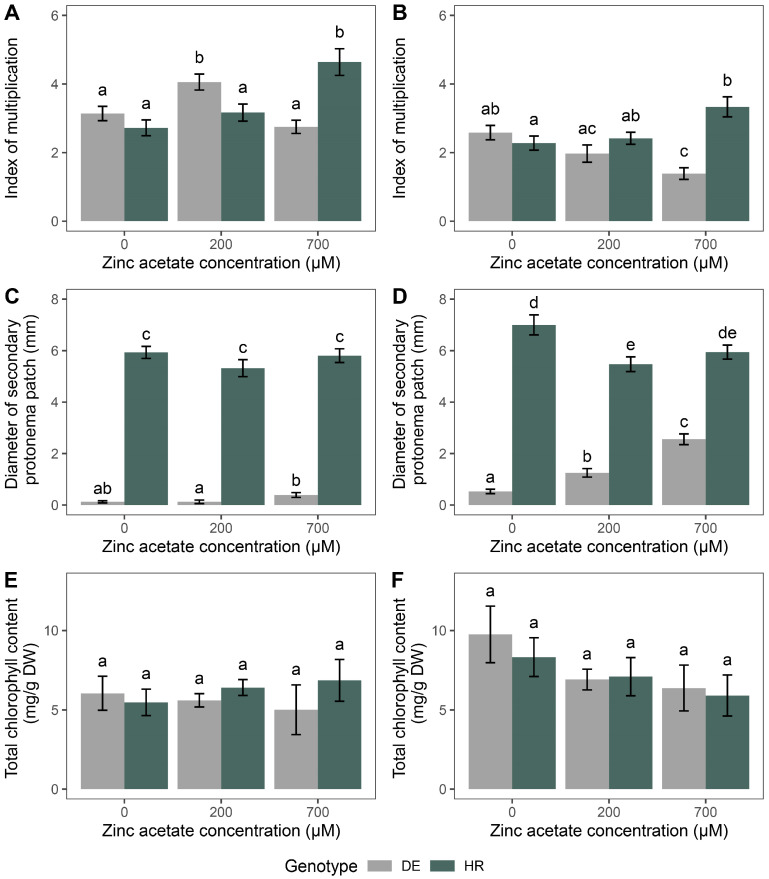
The effects of zinc acetate (0, 200, and 700 µM) at different exposure times on the index of multiplication ((**A**): 2 h, (**B**): 24 h), diameter of the secondary protonema patch ((**C**): 2 h, (**D**): 24 h), and total chlorophyll content ((**E**): 2 h, (**F**): 24 h). Data are presented as mean ± standard error (SE). Letters above the bars indicate statistically significant differences (*p* < 0.05) between experimental groups. Bars sharing the same letter are not significantly different, while bars with different letters represent groups with significant differences.

For both exposure times (2 and 24 h), the index of multiplication parameter significantly increased at 700 µM zinc acetate in the HR genotype compared to the control group (*p* < 0.05), while in the DE genotype, there was no significant increase at the highest applied concentration for the 2 h exposure (Figure 1A,B). However, the highest zinc acetate concentration significantly decreased the index of multiplication of the DE genotype compared to the control group (*p* < 0.05) (Figure 1B). The diameter of the developed secondary protonema patches differed significantly between the two genotypes in the control groups, as well as in any other combination of exposure and zinc acetate concentration (*p* < 0.05) (Figure 1C,D). When exposed to zinc acetate for 2 h, there were no significant changes in secondary protonema patch diameter in any of the genotypes compared to the control groups (Figure 1C). However, changes in the diameter of the secondary protonema patch became evident when plants were exposed to zinc acetate for 24 h, especially in the DE genotype, where the diameter increased significantly with zinc acetate concentration (*p* < 0.05) (Figure 1D), indicating a resource allocation towards protonema development in the DE genotype when exposed to zinc acetate for 24 h. Total chlorophyll content did not change significantly in any of the genotypes when exposed to 200 and 700 µM of zinc acetate for 2 and 24 h (Figure 1E,F).

The main effects of genotype (G) and copper acetate concentration (C) had highly significant effects on the index of multiplication (*p* < 0.001), at both treatment exposures (Table 2). Their interaction (G × C) demonstrated a highly significant effect for shorter exposure (*p* < 0.001) and a moderately significant effect for whole-day exposure (*p* < 0.05) (Table 2). The diameter of the secondary protonema patch was significantly affected by genotype (G), copper acetate concentration (C), and their interaction (G × C) at both times of exposure (*p* < 0.001) (Table 1). Regarding the total chlorophyll content, only the main effect of concentration (C) exhibited a significant effect (*p* < 0.05) for 2 h exposure, while for 24 h exposure, the significant main effects were shown for both genotypes (G) and copper acetate concentrations (C) (*p* < 0.01 and *p* < 0.001, respectively) (Table 2). The interaction of genotype and copper acetate concentration (G × C) had no significant effect on total chlorophyll content at either time of exposure (Table 2).

**Figure 2:**
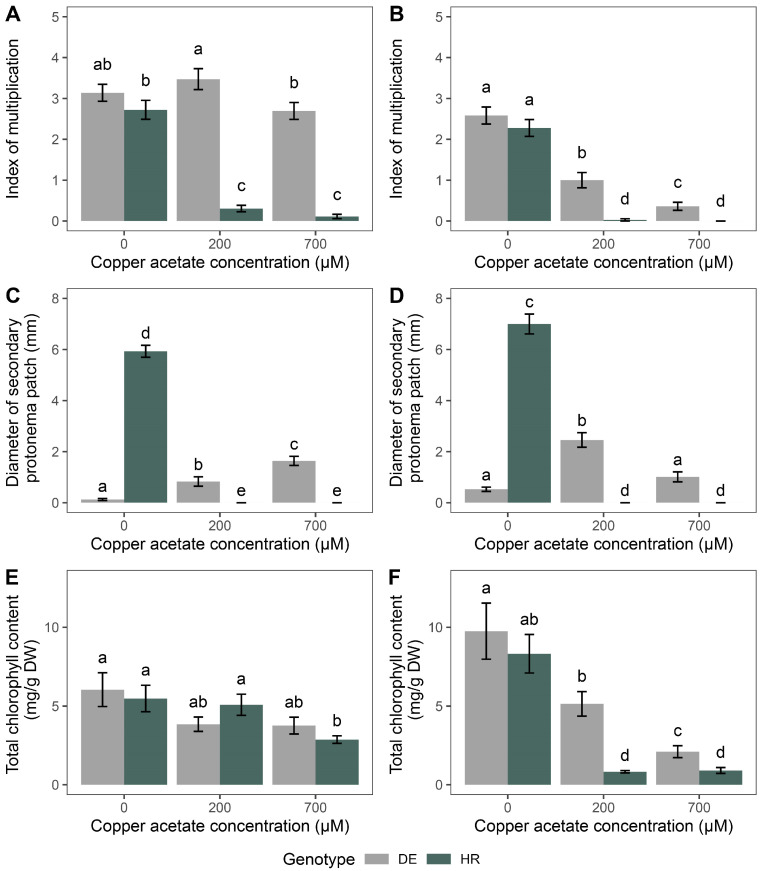
The effects of copper acetate (0, 200, and 700 µM) at different exposure times on the index of multiplication ((**A**): 2 h, (**B**): 24 h), diameter of the secondary protonema patch ((**C**): 2 h, (**D**): 24 h), and total chlorophyll content ((**E**): 2 h, (**F**): 24 h). Data are presented as mean ± standard error (SE). Letters above the bars indicate statistically significant differences (*p* < 0.05) between experimental groups. Bars sharing the same letter are not significantly different, while bars with different letters represent groups with significant differences.

Copper acetate exposure for 2 h, similar to zinc acetate exposure for the same duration, did not change the index of multiplication significantly in the DE genotype relative to the control group (*p* < 0.05); however, a very significant decrease (*p* < 0.05) could be observed in HR genotype (Figure 2A), demonstrating different sensitivity towards copper acetate of the two genotypes. Moreover, when exposed to copper acetate for 24 h, the index multiplication of both genotypes decreased significantly compared to the corresponding control group (*p* < 0.05) (Figure 2B), indicating that prolonged exposure can drastically affect species multiplication. The diameter of the secondary protonema patch was dramatically reduced (*p* < 0.05) when HR genotype plants were exposed to copper acetate at any exposure time (Figure 2C,D). However, in the DE genotype for 2 h exposure, a significant increase in secondary protonema patch with copper acetate concentration was observed (*p* < 0.05), demonstrating completely different responses of the two genotypes to copper acetate (Figure 2C). For 24 h exposure of the DE genotype to copper acetate, a lower concentration (200 µM) significantly increased the diameter of the secondary protonema patch (*p* < 0.05), while the higher concentration (700 µM) exhibited no significant difference compared to the control group (Figure 2D). The total chlorophyll content after 2 h exposure to copper acetate was not significantly different for the DE genotype, but at the highest concentration (700 µM) significant reduction in total chlorophyll concentration compared to the control group was detected for HR accession (*p* < 0.05) (Figure 2E). However, in the case of 24 h exposure, the total chlorophyll content was significantly reduced (*p* < 0.05) for both genotypes relative to the control group, with the absolute values of decrease being more drastic in the HR genotype (Figure 2F), demonstrating higher sensitivity to copper acetate exposure.

A sub-maximal percentage of survival (less than 100% of survived explants per experimental group) for the DE genotype was observed only for 700 µM copper acetate, where documented survival rates were 88.9% and 66.7% for 2 and 24 h exposure, respectively (Table 3). In the case of the HR genotype, sub-maximal survival rates were documented for 24 h exposure at 200 µM copper acetate (97.2%), and in the case of 700 µM copper acetate treatments, the survival rates dramatically declined to 27.8% and 13.9% after 2 and 24 h exposure, respectively (Table 3). Observed survival rates also inferred the higher sensitivity of the HR genotype to copper acetate treatment.

**Figure 3:**
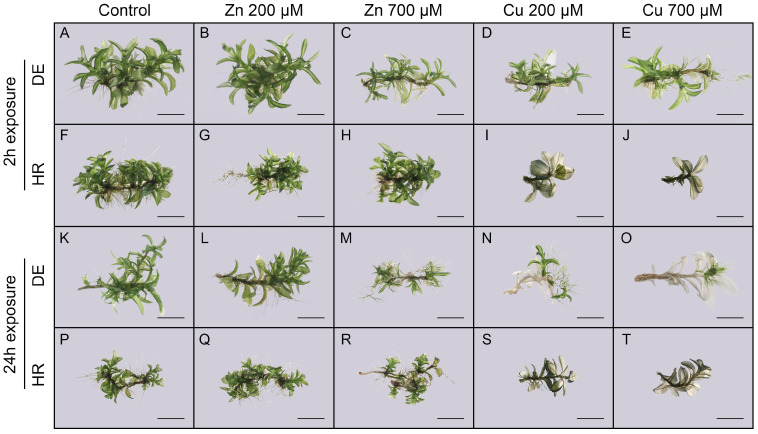
The appearance of *P. eurystomum* plants of the German (DE: (**A**–**E**,**K**–**O**)) and Croatian (HR: (**F**–**J**,**P**–**T**)) genotypes grown on KNOP minimal media, five weeks after treatments with zinc and copper acetate (0, 200, and 700 µM) for 2 and 24 h. The bars represent a size of 2 mm, corresponding to the magnification (0.63×).

In addition, the effects of zinc and copper acetate on the morphogenesis of different *P. eurysyomum* genotypes (DE and HR) were evaluated (Figure 3). Generally, when two genotypes were grown under control conditions, it was evident that the plants from the DE genotype were larger, with bigger phylloids (Figure 3A,F,K,P). The plants of both genotypes exposed to zinc acetate developed normally (Figure 3B,C,G,H,L,M,Q,R), i.e., green gametophores with new branches appeared. Interestingly, slightly underdeveloped plants, with notably less newly developed gametophores and large protonema patches were observed in DE genotype plants when exposed to 700 µM zinc acetate for 24 h (Figure 3M), indicating a slight negative impact of zinc acetate on the DE genotype. When exposed to copper acetate for 2 h, DE genotype plants developed normally (Figure 3D,E), but notable discolorations of the phylloids could be observed, especially at higher copper acetate concentration, together with increased protonema patch size (Figure 3E). When the plants of the DE genotype were exposed to copper acetate for a prolonged time (24 h treatment), the negative effects of copper on plant growth became more evident, especially for plants treated with 700 µM copper acetate. The interesting plant phenotypes could be observed as the initial explant was dead and its apical tissue started to differentiate into secondary protonema (Figure 3O), indicating possible sublethal conditions. The copper acetate exhibited a strong negative effect on the plants of the HR genotype (Figure 3I,J,S,T) where the majority of plants suffered complete phylloid discolorations and did not survive, indicating a severe negative effect of copper.

**Figure 4:**
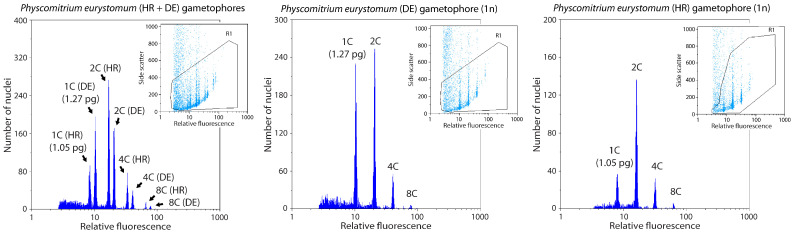
Flow cytometry histograms for two different *P. eurystomum* accessions (German—DE, and Croatian—HR). FCM record of mixed sample (**left**), for which both DE and HR genotypes were prepared simultaneously for FCM. 1C—haploid, 2C—diploid, 4C—tetraploid, and 8C—octaploid nuclei.

In gametophytes of different genotypes of *P. eurystomum*, flow cytometry was used to measure the different types of nuclei (Figure 4). The 2C nuclei were the most abundant in gametophytes of both genotypes and in both, the three gradually declining peaks were identified, indicating endoreduplication. The recorded genome sizes in the two genotypes were different, with the DE genotype having 1.27 pg/1C compared to the HR genotype with 1.05 pg/1C.

## 3. Discussion

Considering both, zinc and copper acetate treatments, the genotype and concentration play critical roles in influencing plant responses. The effects of treatment concentrations on the morphogenesis of the species, assessed using the index of multiplication and the diameter of the secondary protonema patch, were shown to be genotype dependent. However, the opposite was observed in the case of total chlorophyll content, where no significant effects were observed in the case of zinc acetate treatments, while in the case of copper acetate treatments, no significant interaction was observed, implying that the effect of copper acetate concentration on total chlorophyll content does not depend on the genotype. Comparing the plant phenotypes, especially in the case of copper acetate treatment, it can be detected that different genotypes have different sensitivity to treatments. It seems that in general, zinc acetate has a slightly positive effect on morphogenesis of the HR genotype, particularly in the case of 2 h treatments, and a slightly negative effect on the DE genotype. Even though the negative effect of copper acetate on *P. eurystomum* is evident, the difference in genotype sensitivity towards the treatment suggests that the DE genotype is more resilient. Furthermore, the observed difference in genome size (in favor of the DE genotype), likely associated with ploidy level difference, could provide the answer to its increased resilience to copper acetate, as it was previously shown that the anatomical and physiological changes generated by either natural or artificial polyploidization could increase tolerance to abiotic stress [16]. Furthermore, it was also shown that polyploidization in naturally occurring hyperaccumulators could lead to their range expansion [17].

The induction of protonemal development as a response to stressors can occur in moss species [18], as it happened in the case of DE genotype after 24 h exposure to zinc acetate. On the other hand, the opposite was observed in the case of copper acetate treatments, probably because of the higher toxicity of copper, and such a decrease in protonema patch diameter was previously observed in other moss species in the case of copper treatments [19]. Furthermore, a reduction in the number of newly formed shoots (index of multiplication) was also common when moss species were under stress conditions [20], especially PTE stress [21]. Also, increased PTE concentrations, particularly copper, could dramatically reduce the survival rates of the mosses [21,22]. Similarly, copper toxicity was well known to induce chlorosis in mosses [13], as well as in tracheophytes [23].

It seems that different mosses respond differently towards zinc stress evaluated by total chlorophyll content and that the response is the dosage and exposure length related [24,25], even a slightly positive correlation could be found between the zinc concentration and chlorophyll content, for example, in *Pleurozium schreberi* (Brid.) Mitt. [26]. A slight increase in total chlorophyll content was observed in this study in the case of 2 h zinc acetate treatments in the HR genotype, although this is not significant. On the other hand, copper’s effects on chlorophyll content in mosses were undoubtedly negative [24,26]. Reduced photosynthetic rates of copper-exposed mosses were also well documented, for example, in *Rhytidiadelphus squarrosus* (Hedw.) Warnst. [27] starting at low concentrations, a slight reduction of photosynthetic function was also observed in the same study in zinc-treated plants, but only at very high concentrations (0.1 M).

The mechanism of this negative effect of copper on morphogenesis and chlorophyll content can be explained by the high affinity that copper and other PTEs have for sulfhydryl groups (-SH) of proteins, which can lead to the inhibition of enzyme activity and changes in protein conformations [28]. Also, it can degrade the phospholipid structure, altering membrane structure function [29]. Copper is believed to be replacing the cofactors (such as Mg^2+^, Zn^2+^, and Fe^2+^) in photosynthetic pigments and chloroplasts making them unstable or inactive, which leads to their degradation and a decrease in photosynthetic activity [30]. For illustration, it is well known that copper ions replace the Mg^2+^ in the aromatic porphyrin ring system resulting in the destruction of chlorophyll and, consequently, a reduction in chlorophyll content [31]. Exposure of plants to high concentrations of copper reduces absorption and homeostasis of other mineral nutrients [32,33], probably through the mechanism of ion competition. Furthermore, PTEs can cause disturbance in biochemical and physiological processes in plant cells through the alteration of the nitrogen cycle [34]. For example, in mosses, it was documented that PTEs could induce a decrease in nitrogen incorporation to amino acids [35], or inhibition of nitrate reductase [36].

In a phylogenetically related species, model moss *Physcomitrium patens* (Hedw.) Mitt. treated with zinc and copper, less toxic zinc was more accumulated in the moss tissues compared to copper, and, interestingly, metal contents of protonemata cells were higher than those of phylloid cells as the authors indicated that protonemata cells seemed to be more tolerant [14]. Furthermore, copper is shown to induce the development of secondary protonema in *Scopelophila cataractae* (Mitt.) Broth. as it did in the DE genotype in this study, enabling the survival of the species and demonstrating different tolerance to copper of gametophore and protonema tissues [37]. Interestingly, the DE genotype does not develop protonema patches in control groups, or they are quite small and they get larger in copper-treated plants, contrary to the HR genotype that develops large protonema patches in control groups whose development is severely hindered in copper-treated groups.

The results obtained in this study clearly show that different genotypes respond differently to PTE treatments, but more in-depth studies of the genotypes are needed for further elucidation. It was previously confirmed that the DE genotype of the tested *P. eurystomum* is most probably of hybrid origin [38]. However, the HR genotype could be of non-hybrid origin or the result of another hybridization event with other species from the *Physcomitrella*–*Physcomitrium* complex. This may also lead to differences in physiological traits and survival rates. Indeed, the two genotypes grown under the same axenic laboratory conditions developed rather differently. In fact, the HR genotype was smaller and frequently produced sporophytes with viable spores [39], while the DE genotype was somewhat larger and produced neither sexual organs nor sporophytes.

## 4. Materials and Methods

### 4.1. Plant Material and In Vitro Cultivation

Two accessions of *P. eurystomum* from Germany and Croatia were used in this study. German accession originates from Neukirch, Baden-Württemberg (coll. Schäfer-Verwimp 28 August 2001, No. 40048; 48.0167° N, 8.1833° E). The Croatian accession (HR genotype) of the species originates from the population located on the shores of Bilje Lake, Baranja region of Croatia (45.5901° N, 18.7360° E, altitude 84 m a.s.l.). The Croatian collection took place on 23 September 2017, leg. A. Rimac, N. Koletić, N. Vuković, det. A. Rimac (voucher ZA49325). The sporophyte capsules, cleaned from mechanical impurities, were used to establish in vitro cultures of the genotype (for details see [39]), while the German accession (DE genotype) was obtained as an in vitro culture established at the University of Marburg, Germany [40].

Plants of both genotypes were grown and propagated on KNOP minimal medium (250 mg L^−1^ KH_2_PO_4_, 250 mg L^−1^ MgSO_4_, 250 mg L^−1^ KCl, 100 mg L^−1^ Ca(NO_3_)_2_, 2.5 mg L^−1^ FeSO_4_×7H_2_O, 8 g L^−1^ agar) [41] in order to obtain the sufficient biomass needed for the experiments. The pH value of the media was adjusted to 5.8 before autoclaving at 121 °C for 45 min at 0.067 MPa. Plants were grown under sterile conditions at constant temperature (18 ± 2 °C) and humidity (60–70%) in a long-day light regime (16 h light/8 h dark). Illumination was provided by cool white fluorescent tubes (Tesla, Pančevo, Serbia) with a photon flux density of 40 µmol m^−2^ s^−1^. In the experiment, six-millimeter-long, single gametophores were used as initial explants and each was planted vertically into the medium. All the explants were used one month after subcultivation.

### 4.2. Experimental Design

The effects of zinc and copper acetate (supplied by Sigma Aldrich, Steinheim, Germany) on the development of different genotypes of *P. eurystomum* were assessed. Plant explants were treated with 200 and 700 µM solutions of zinc and copper acetate, exposing them to sterilized solutions in the Magenta boxes for 2 and 24 h, shaking at 110 rpm on an orbital shaker (BioSan PSU-20i, Riga, Latvia) at room temperature. In control groups, plants were treated with distilled sterile water for the equivalent time. After the treatment, gametophores were rinsed in sterile distilled water, drained of excess water, and planted on KNOP minimal media in sterile conditions under the laminar hood, for a recovery period in duration of five weeks, after which the results of the experiment were assessed. Each experimental group consisted of 36 individual explants (n = 36).

### 4.3. Morphogenetic and Physiological Parameters

The morphogenesis of the species was characterized using standard morphogenesis parameters such as the index of multiplication, i.e., the number of newly formed shoots per initial explant and the diameter of the secondary protonema patch [42,43]. Moreover, the survival percentage per experimental group was also assessed.

The photosynthetic pigments were extracted in 1.5 mL of 96% (*v*:*v*) ethanol from 15 mg of moss gametophores per sample per experimental group. The samples were incubated at 70 °C for 10 min and centrifuged (Tehtnica Centric 200R, Zelenzniki, Slovenia), after which the supernatant was transferred to a microtiter plate, and the sample absorbance was measured at two wavelengths (648 and 664 nm) using the Multiscan SkyThermo Scientific plate reader (Waltham, MA, USA). The total chlorophyll content in the moss gametophores was then calculated according to the equation: C(a + b) = 5.24 × A664 + 22.24 × A648 [44] and expressed as mg g^−1^ dry weight. Each experimental group consists of five biological replicates (n = 5), measured in triplicates.

The photographs of the explants were taken using a Leica MZ stereomicroscope (Leica MZ 7.5 Bi-Optic Inc., Santa Clara, CA, USA).

### 4.4. Flow Cytometry Analysis

The nuclei samples for flow cytometry determination of nuclear DNA content were prepared according to a standard chopping method as performed earlier [45] but with the addition of a reference standard. Briefly, green gametophores of mosses and approximately equally sized leaf parts of the reference standard *Bellis perennis* L. [46] were used. The DNA content of the 2C value is 3.134 pg. Note that the genome size of *B. perennis* was recalculated, as elaborated previously [47,48]. The samples were chopped with a razor blade in 1 mL of general-purpose buffer (GPB buffer, [49]) in a Petri dish. The suspension was then filtered through a 42 μm nylon filter and the final liquid nuclei isolate was supplemented with RNAase (in a final concentration of 50 μg mL^−1^), propidium iodide (50 μg mL^−1^), and β-mercaptoethanol (2 μLmL^−1^). Samples were analyzed using a CyFlow ML cytometer equipped with a green laser of 320 nm wavelength (Partec GmbH, Münster, Germany). The resulting histograms of relative fluorescence were displayed on a linear scale. The peaks were manually ranged on FCM histograms and represented the number of nuclei of respective ploidy levels. Mosses are well-known polysomatic organisms, and their thalli contain mosaics of different ploidy levels (1C, 2C, 4C…) due to a common endopolyploidization [50]. Mean values of moss and reference standard peaks were used to calculate the DNA content of 1C or 2C peaks, as described previously [51]:

DNA content of the sample = DNA content of the standard × [(the sample peak mean)/(the standard peak mean)].

Finally, the DNA content (1C or 2C value) of the species was calculated as an average of the three independent FCM measurements per accession, i.e., each accession was measured three times, on different days.

### 4.5. Statistical Analysis

Statistical analysis was carried out using the R programming language (v. 4.3.1) [52]. Preliminary data exploration consisted of the Levene test for homogeneity of variance and the Shapiro–Wilk normality test. Consequently, nonparametric Aligned Rank Transform (ART) analysis was chosen, as not all experimental groups met the assumptions of normality and homoscedasticity.

The nonparametric factorial ANOVA was conducted using the ART procedure [53,54]. The ART analysis was applied to each parameter and exposure time for zinc and copper acetate, employing the “ARTool” R package [55]. Factorial models were built using the “art” function, and the significance of main effects and interactions was assessed with the “ANOVA” function. Post hoc contrast tests were carried out using the “art.con” function from the same package.

## 5. Conclusions

Zinc and copper influence the survival and development of the rare and threatened moss *P. eurystomum*. In general, Zn acetate seems to have a slightly positive effect on the morphogenesis of the HR genotype, while it has a slightly negative effect on the DE moss genotype. As expected, copper showed a strong negative effect on moss morphogenesis and physiological parameters, especially when compared to zinc treatments. Furthermore, the DE genotype was less sensitive to copper compared to the HR accession. The genetic structure, as inferred by genome size and the origin of this funarioid species, which is frequently exposed to hybridization, could provide an explanation for the differences in the PTE stress resilience of this species.

## Figures and Tables

**Table 1:** Summary results of the factorial analysis, evaluating the effect of genotype (G), zinc acetate concentration (C), and their interaction (G × C) on the index of multiplication, diameter of secondary protonema patch, and total chlorophyll content for different exposure times (2 and 24 h).

Parameter	Time	G	C	G × C
Index of multiplication	2 h	0.1329	5.2346 **	14.4338 ***
	24 h	9.83698 **	0.77777	12.33015 ***
Diameter of secondary protonema patch	2 h	690.2289 ***	7.6139 ***	2.9338
	24 h	556.978 ***	9.338 ***	18.761 ***
Total chlorophyll content	2 h	1.43918	0.26049	0.70407
	24 h	0.305874	0.332833	0.088626

The values represent the F values with the asterisks indicating the corresponding level of statistical significance, ** *p* < 0.01, *** *p* < 0.001.

**Table 2:** Summary results of the factorial analysis, evaluating the effect of genotype (G), copper acetate concentration (C), and their interaction (G × C) on the index of multiplication, diameter of secondary protonema patch, and total chlorophyll content for different exposure times (2 and 24 h).

Parameter	Time	G	C	G × C
Index of multiplication	2 h	206.703 ***	39.088 ***	33.927 ***
	24 h	19.6348 ***	165.9156 ***	3.8402 *
Diameter of secondary protonema patch	2 h	102.78 ***	177.32 ***	906.67 ***
	24 h	72.168 ***	137.309 ***	677.689 ***
Total chlorophyll content	2 h	0.0032258	5.554 *	1.0518
	24 h	8.7461 **	49.7198 ***	3.2631

The values represent the F values with the asterisks indicating the corresponding level of statistical significance, * *p* < 0.05, ** *p* < 0.01, *** *p* < 0.001.

**Table 3:** Survival percentage of two *P. eurystomum* genotype explants (DE and HR) exposed to different concentrations of zinc and copper acetate (200 and 700 µM) for 2 and 24 h.

Treatment	Time	DE Genotype	HR Genotype
Control	2 h	100%	100%
	24 h	100%	100%
Zinc acetate 200 µM	2 h	100%	100%
	24 h	100%	100%
Zinc acetate 700 µM	2 h	100%	100%
	24 h	100%	100%
Copper acetate 200 µM	2 h	100%	100%
	24 h	100%	97.2%
Copper acetate 700 µM	2 h	88.9%	27.8%
	24 h	66.7%	13.9%

## Data Availability

All the data are available by authors upon request.
